# Reduction of Sphingosine Kinase 1 Phosphorylation and Activity in *Plasmodium*-Infected Erythrocytes

**DOI:** 10.3389/fcell.2020.00080

**Published:** 2020-03-03

**Authors:** Raj Kumar Sah, Soumya Pati, Monika Saini, Pon Arunachalam Boopathi, Sanjay Kumar Kochar, Dhanpat Kumar Kochar, Ashis Das, Shailja Singh

**Affiliations:** ^1^Special Centre for Molecular Medicine, Jawaharlal Nehru University, New Delhi, India; ^2^Department of Life Sciences, School of Natural Sciences, Shiv Nadar University, Greater Noida, India; ^3^Department of Biological Sciences, Birla Institute of Technology and Science, Pilani, India; ^4^Department of Medicine, Sardar Patel Medical College, Bikaner, India; ^5^Sardar Patel Medical College, Bikaner, India

**Keywords:** erythrocytes, *Plasmodium falciparum*, *Plasmodium vivax*, sphingosine kinase-1, sphingosine-1-phosphate, complicated malaria, uncomplicated malaria, thrombocytopenia

## Abstract

Sphingosine-1-phosphate (S1P), a bioactive lipid mediator is involved in an array of biological processes and linked to pathological manifestations. Erythrocyte is known as the major reservoir for S1P as they lack S1P-degrading enzymes (S1P lyase and S1P phosphohydrolase) and harbor sphingosine kinase-1 (SphK-1) essential for sphingosine conversion to S1P. Reduced S1P concentration in serum was correlated with disease severity in patients with *Plasmodium falciparum* and *Plasmodium vivax* infections. Herein, we aimed to identify the underlying mechanism and contribution of host erythrocytes toward depleted S1P levels in *Plasmodium*-infected patients vs. healthy individuals. The level and activity of SphK-1 were measured *in vitro* in both uninfected and cultured *P. falciparum*-infected erythrocytes. Infected erythrocytes demonstrated a significant decrease in SphK-1 level in a time-dependent manner. We found that 10–42 h post invasion (hpi), SphK1 level was predominantly reduced to ∼50% in rings, trophozoites, and schizonts compared to uninfected erythrocytes. We next analyzed the phosphorylation status of SphK-1, a modification responsible for its activity and S1P production, in both uninfected control and *Plasmodium*-infected erythrocytes. Almost ∼50% decrease in phosphorylation of SphK-1 was observed that could be corroborated with significant reduction in the production and release of S1P in infected erythrocytes. Serum S1P levels were studied in parallel in *P. falciparum* (*N* = 15), *P. vivax* (*N* = 36)-infected patients, and healthy controls (*N* = 6). The findings revealed that S1P concentration was significantly depleted in uncomplicated malaria cases and was found to be lowest in complicated malaria and thrombocytopenia in both *P. falciparum* and *P. vivax*-infected groups (^∗∗^*p* < 0.01). The lower serum S1P level could be correlated with the reduced platelet count defining the role of S1P level in platelet formation. In conclusion, erythrocyte SphK-1 and S1P levels were studied in *Plasmodium*-infected individuals and erythrocytes that helped in characterizing the complications associated with malaria and thrombocytopenia, providing insights into the contribution of host erythrocyte biology in malaria pathogenesis. Finally, this study proposes the use of S1P and its analog as a novel adjunct therapy for malaria complications.

## Introduction

According to World Health Organization (WHO) report, an estimate of 219 million malaria cases have been detected with 435,000 number of deaths (World Health Organization [WHO], 2018). *Plasmodium falciparum*, being the most virulent causative agent of malaria, accounts for most of these cases (Bloland and Bloland, 2001). Elucidating the biology of *P. falciparum* is of prime importance for understanding its pathology and clinical manifestations in humans. During malaria infection, the parasite acquires the host erythrocyte cell lipids, responsible for various lipid-dependent cell signaling pathways including protein trafficking and hemoglobin degradation (Gulati et al., 2015). Synthesis of lipids and lipid-dependent pathways have been shown to be essential for the intracellular (IC) growth of human malaria parasites (Bobenchik et al., 2011). Pathogenesis of malaria is a complex phenomenon that is mediated through both the parasite and host-related factors. It is noteworthy that the cytoadhesion of infected erythrocytes to vascular endothelial cells as well as the dysregulated production of inflammatory cytokines are considered to be the major reasons underlying the systemic inflammatory illness linked to disease severity in malaria that leads to death of the patient (Pongponratn et al., 1991; Lyke et al., 2004). Sphingosine-1-phosphate (S1P), a signaling biolipid has been regarded as a key mediator for regulating the vasculature of the endothelial cell lining that maintains vascular barrier function via a series of molecular events triggered by S1P docking to its receptor S1P1. This leads to a sequel of molecular events, including; subsequent activation of Rho GTPases, cytoskeleton reorientation, and formation of adherens and tight junction assembly, etc. (Camerer et al., 2009; Snider et al., 2010; Finney et al., 2011). S1P is also known to be involved in various biological processes including immune response (Rivera et al., 2008), bone marrow cell trafficking (Kong et al., 2014), and cell survival and proliferation (Inniss and Moore, 2007). Given the diverse roles of S1P, various cell types have been identified as production and storage house for S1P including erythrocytes (Hänel et al., 2007), platelets (Schaphorst et al., 2015), endothelial cells (Pham et al., 2010), mast cells (Jolly et al., 2004), and macrophages (Xiong et al., 2013). However, erythrocytes have been considered as the main repository for S1P in the blood plasma (Bode et al., 2010). Several reasons have been held responsible for the elevated S1P content in these cells including high sphingosine kinases (SphK) activity, lack of S1P-degrading enzymes (S1P lyase and S1P phosphohydrolase), and its capability to import sphingosine from extracellular (EC) environment (Zhou and Saba, 1998; Le Stunff et al., 2007). Surprisingly, the role of multi-faceted S1P in erythrocytes and its effects on the physiology of the cells are yet to be elucidated. Particularly, the sphingolipid metabolites, ceramide (Cer) and S1P, have emerged as a new class of potent bioactive molecules. Ceramide can be generated by *de novo* or hydrolysis of membrane sphingomyelin by sphingomyelinase (SMase), and subsequently metabolized by ceramidase to generate sphingosine (Sph), which in turn is phosphorylated by sphingosine kinases 1 and 2 (SphK1, 2) to generate S1P, a pleotropic bioactive lipid mediator. Both ceramide and S1P regulate cellular responses to stress, with generally opposing effects (Maceyka et al., 2012; Nakahara et al., 2012). Among these enzymes, SphK-1 is localized to the cytosol, while SphK-2 is found to be present in the nucleus (Pitson et al., 2003; Hait et al., 2009). Erythrocytes harbor only SphK-1 that is the main enzyme responsible for the production of S1P (Hänel et al., 2007; Bode et al., 2010). Phosphorylation of host SphK-1 acts as a key-regulating factor underlying its activity. Existing evidence suggested that phosphorylation of serine 225 in SphK-1 enables its binding to phosphatidylserine, membrane relocalization, followed by membrane-associated activity (Pitson, 2011). Notably, in the case of infectious diseases like leishmaniasis, a reduced level of phosphorylated SphK-1 with deregulated S1P signaling was reported that could be corroborated with the disease pathology (Arish et al., 2018). Earlier reports have also investigated the serum levels of circulating S1P in humans during various disease conditions correlating it to the disease severity (Maceyka et al., 2012). However, recent studies have highlighted an essential role of S1P signaling in thrombopoiesis wherein S1P interacts with its receptors, S1P_1_ and S1P_4_, expressed in megakaryocytes for platelet production and release to the circulation Golfier et al., 2010; Zhang et al., 2012). Modulation of circulating S1P gradient between vascular and non-vascular compartments might result in destabilized S1P signaling-mediated attenuated thrombopoiesis (Schwab et al., 2005; Hla et al., 2008). It is also noteworthy that circulating S1P has been linked to severity in clinical manifestations during malaria (Punsawad and Viriyavejakul, 2017). With this background, we aimed to study the level and activity of SphK-1 as well as the level of S1P during *in vitro* and *in vivo* malaria infection. The levels and activity of SphK-1 were measured *in vitro* in both uninfected and cultured *P. falciparum*-infected erythrocytes. Infection of erythrocytes led to decreased levels of SphK-1 post invasion in a time-dependent manner. Following 10–42 hpi, the SphK-1 levels were significantly lowered in ring, trophozoite, and schizont stages compared to uninfected erythrocytes. To further analyze SphK-1 activity, we evaluated SphK-1 phosphorylation and S1P production, in both uninfected and *Plasmodium*-infected erythrocytes. A prominent drop in SphK-1 phosphorylation was observed that was then corroborated with significant reduction in production and release of S1P in infected erythrocytes. In addition, serum S1P levels were estimated in parallel in *P. falciparum* (*N* = 15)-, *P. vivax* (*N* = 36)-infected patients, and healthy controls (*N* = 6) to understand the clinical relevance of SphK-1 activity linked to S1P production. Though the serum S1P level was found to be significantly reduced in uncomplicated malaria cases, it was found to be lowest in complicated malaria and thrombocytopenia in both *P. falciparum* and *P. vivax* malaria groups (^∗∗^*p* < 0.01). This depleted level of serum S1P can be correlated with reduced platelet count defining the possible role of S1P level in platelet formation, thus providing insights into the contribution of host-SphK-1 signaling in malaria pathogenesis. Taken together, we present the experimental proof of decreased S1P levels during malaria infection associated with thrombocytopenia and chronic malaria because of altered SphK-1 activity. Finally, this study suggests the use of S1P and its analog as a novel adjunct therapy for malaria complications.

## Materials and Methods

### *In vitro* Culture of *P. falciparum*

*Plasmodium falciparum* 3D7 strain was cultured using O+ human erythrocytes, under mixed gas environment (5% O_2_, 5% CO_2_, and 90% N_2_) as described previously (Trager and Jensen, 1976). The culture media was composed of RPMI 1640 (Invitrogen, Carlsbad, CA, United States), supplemented with 27.2 mg/l hypoxanthine (Sigma-Aldrich, St. Louis, MO, United States), 2 g/l sodium bicarbonate (Sigma-Aldrich, St. Louis, MO, United States), and 0.5 g/l Albumax I (Gibco, Grand Island, NY, United States). Human erythrocytes (O+) as well as the serum of healthy volunteers were procured from the blood bank and used only for research purposes. Synchronous development of the erythrocytic stages of a human malaria parasite, *P. falciparum*, in culture was accomplished by suspending cultured parasites in 5% D-sorbitol and subsequent reintroduction into culture (Lambros and Vanderberg, 1979).

### Immunofluorescence Assay for SphK-1 Level

Immunofluorescence assay (IFA) was performed to detect the level of SphK-1 in *P. falciparum* 3D7. Thin smears of ring, trophozoite, and schizont stage parasites were made on glass slides, air dried, and fixed with methanol (ice cold, 100%) for 30 min at −20°C. Smears were then blocked with 3% (*w*/*v*) bovine serum albumin (BSA) in phosphate buffer saline (PBS) blocking buffer (pH 7.4) for 30 min at room temperature (RT). After blocking, slides were incubated with anti-SphK-1 (1:1,000 in blocking buffer; Invitrogen, Carlsbad, CA, United States) at RT for 1 h. Slides were washed with 0.05% Tween-20 in PBS (PBST) two times followed by washing with PBS and incubated with an Alexa Fluor 594 conjugated goat anti-rabbit IgG (1:500 dilution; Molecular Probes, United States) at RT for 1 h. After removal of unbound antibodies with three PBS washes, slides were mounted with Pro Long Gold antifade reagent (Invitrogen, Carlsbad, CA, United States) with coverslips and viewed on a Nikon A1-R confocal microscope using a 100× (oil) objective, and acquired images were processed via NIS-Elements software. Mean fluorescent intensity (MFI) was determined for a single cell by measuring fluorescent intensity corresponding to Sphk-1 (red signal) for uninfected erythrocyte and infected erythrocyte and plotted as bar graph showing the MFI at a single-cell level. All imaging parameters were kept the same during acquisitions.

### Immunoblotting of SphK-1 for Its Activity and Level

Immunoblotting of SphK-1 and Phospho-SphK-1 (Ser225) was performed in parasite-infected and uninfected erythrocytes to analyze SphK-1 level and activity. Total cell pellets of parasite-infected and uninfected erythrocytes were re-suspended in RIPA buffer [100 mM phosphate buffer (pH 7.2), 150 mM sodium chloride (NaCl), 1% NP-40, 0.5% sodium deoxycholate, 0.1% sodium dodecyl sulfate (SDS), 50 mM ethylenediaminetetraacetic acid (EDTA), and 1× protease inhibitor cocktail (PIC)]. Equal amounts of each sample was boiled with 2× Laemmli buffer and separated on a 10% sodium dodecyl sulfate polyacrylamide gel electrophoresis (SDS–PAGE). The fractionated proteins were transferred from gel onto the PVDF membranes (Millipore) using the Trans-blot Turbo transfer system and blocked with 5% skim milk in Tris buffer saline (TBS; blocking buffer) for 1 h at 4°C. The blots were washed twice with 0.1% Tween-20 in TBS (TBST) followed by TBS and incubated overnight at 4°C with anti-SphK-1 (1:3,000) and anti-Phospho-SphK-1 (Ser225; 1:1,000; Invitrogen, Carlsbad, CA, United States) rabbit antibodies in blocking buffer (TBS, 1% skim milk). GAPDH present in the lysates was used as a loading control and probed with anti-GAPDH (1:10,000; Invitrogen, Carlsbad, CA, United States) mouse antibody. Later, the blots were rinsed in TBST three times, 5 min each, and in Tris-buffered saline (TBS) for 5 min and incubated for 1 h with appropriate secondary antibodies anti-rabbit and anti-mouse (1:10,000) conjugated to HRP; after being rinsed in TBST three times, 5 min each, and in TBS for 5 min, target proteins were visualized using the Clarity Western ECL substrate (Bio-Rad). The intensity of each band was quantified with ImageJ software and defined as Arbitrary Unit (AU) and plotted as bar graph.

### Extraction of Lipids for Measuring S1P

For S1P measurement, an equal number of synchronous parasitized erythrocytes at 7–8% parasitemia and uninfected erythrocytes were taken. Collected cell pellets and supernatant were employed for lipid extraction as reported previously (Zhao and Xu, 2010). Pellets were resuspended in 100 μl of H_2_O and transferred to 900 μl of methanol. To quantify S1P in supernatant, a 1:15 ratio of methanol was taken. After vortexing and centrifugation at 10,000 × *g* for 5 min at RT, methanol extracts were removed to a new glass tube. After evaporation by N_2_, dried lipids were resuspended in 200 μl of methanol. Extracted lipid samples were subjected to liquid chromatography–mass spectrometry (LC/MS) analysis. We used a Waters Acquity H-Class UPLC system (Waters, Milford, MA, United States). Chromatographic separation was achieved on an Acquity BEH C18, 1.7 μm, 75 × 2.1 mm column (Waters, Manchester, United Kingdom). The mobile phase consisted of 0.1% formic acid solvent (A) and acetonitrile (B). The initial gradient condition was 90% A and 10% B, 80 and 20% for 2 min, 50 and 50% for 3 min, 20 and 80% for 1 min, 10 and 90% for 2 min, and then linearly changed to 34% B over 8 min and turned back to initial condition of 90% A and 10% B and washed up to 15 min. The column temperature was adjusted at 35°C. The flow rate was 0.3 ml/min, and the injection volume was 5 μl. Mass spectrometry was performed in negative electrospray mode using a high-resolution mass spectrometer SYNAPT G2 S HDMS (Waters, Manchester, United Kingdom) with a TOF-detector with linear dynamic range of at least 5,000:1. The desolvation gas (45°C, 647.0 l/h) and the nebulizer gas (6.0 bar) were nitrogen. The cone gas had a flow of 52 l/h. The capillary voltage was 2.52 kV and the source temperature 90°C. The analyzer mode was set at “resolution” and the dynamic range at “extended.” The mass spectra were acquired over the range of 100–1000 Da with a spectral acquisition rate of 0.1 s per spectrum. For the S1P measurement by enzyme-linked immunosorbent assay (ELISA; MyBioSource, San Diego, CA, United States) method, parasite-infected and uninfected erythrocytes were centrifuged to collect the supernatant. Pelleted erythrocytes were washed and lysed by multiple freeze–thaw cycles. EC (extra cellular) S1P was measured through the supernatant, whereas the lysed erythrocytes were used to measure IC (intra cellular) S1P level. Erythrocyte supernatant, lysate, and patient serum samples were added to the micro ELISA plate wells separately, pre-coated with S1P-specific antibody for 90 min at 37°C. A biotinylated detection antibody specific to human S1P was added and incubated for 1 h at 37°C. After washing, avidin–horseradish peroxidase (HRP) conjugate was added successively to each microplate well and incubated for 30 min at 37°C. The wells were washed to remove the unbound components followed by the addition of substrate solution to each well. Only those wells that contained S1P, biotinylated detection antibody, and avidin–HRP conjugate appeared blue in color. The enzyme–substrate reaction was terminated by the addition of stop solution turning the reaction color to yellow. The optical density (OD) proportional to the S1P level was measured spectrophotometrically at a wavelength of 450 nm.

### Ratio Calculation Between SphK-1/phosphorylated SphK-1 (pSphK-1)

Intensity AU (IAU) for Sphk-1 and pSphk-1 in both uninfected and infected erythrocytes were normalized with band intensities of loading control GAPDH. The relative ratio of normalized band intensity for SphK-1 and pSphK-1 was determined. Finally, the ratio of pSphK-1 to SphK-1 intensity was plotted as AU.

### Study Site and Procedures

The collection of patient serum samples was done at the Department of Medicine, Sardar Patel Medical College and associated group of Hospitals, Bikaner, India. Bikaner is a part of the Thar Desert situated in the northwest part of India near the Indo–Pak border and is a hypoendemic region for malaria. This prospective study was conducted on admitted adult patients of malaria in whom the diagnosis was done by peripheral blood smears (PBFs) and rapid diagnostic tests (RDTs). After thorough clinical and laboratory investigations, the categorization of severe malaria and treatment was done according to the WHO guidelines (Trampuz et al., 2003). The final confirmation of species diagnosis was done by polymerase chain reaction (PCR) examination-based validation. The hospital ethical committee [Department of Medicine, Sardar Patel Medical College and associated group of Hospitals, Bikaner, India; sample collection number, F. (Acad) SPMC/2003/2395] was obtained against the approval and written consent by patients. Adult patients of malaria with severe manifestations and evidence of asexual phase of malaria parasite in PBF and/or positive RDT along with positive PCR evident of malaria were set as the selection criteria, whereas patients who refused to give the written consent or had evidence of other concurrent illness not included in the study were used as exclusion criteria.

### RDT- and PCR-Based Detection of *P. falciparum* and *P. vivax* in Clinical Samples

Diagnostic methods used for the detection of malaria parasites were conventional thick and thin PBF stained with Giemsa stain (Sigma-Aldrich, St. Louis, MO, United States) and microscopically examined under oil immersion. The slide was considered negative when there were no parasites in the 100 high-power field object. The RDTs were based on the detection of specific *Plasmodium* antigen, lactate dehydrogenase (OptiMal test; Diamed AG, Cressier sur Morat, Switzerland), and histidine-rich protein-2 (Falcivax test; Zephyr Biomedical System, Goa, India). The final categorization of *P. vivax*, *P. falciparum*, or mixed infection was done by PCR examination of all the patients having severe manifestations. The PCR studies were targeted against the 18S ribosomal RNA gene of the parasite and used one genus-specific 5′-primer and two species-specific 3′-primers in the same reaction mixture or a nested PCR targeted against the 28S ribosomal RNA gene of the parasites (Das et al., 1995; Kochar et al., 2005; Pakalapati et al., 2013a, b). The amplified product was run on 1% agarose gel, and the desired band was observed.

### Clinical Investigations

Laboratory investigations done in all the patients of severe malaria included complete blood count, platelet count, bleeding time, clotting time, blood glucose, blood urea, serum creatinine, serum bilirubin (conjugated and unconjugated), serum aspartate aminotransferase (AST), serum alanine aminotransferase (ALT), serum alkaline phosphatase, complete urine analysis, electrocardiogram, and appropriate blood test to rule out typhoid fever (typhi dot test), leptospirosis, dengue infection (differential detection of IgG and IgM antibodies), and human immunodeficiency virus (HIV). Depending upon the clinical situation, other tests included skiagram of chest, serum electrolytes, and arterial blood gas analysis for acute respiratory distress syndrome (ARDS); fundus examination, cerebrospinal fluid (CSF) examination, computerized tomography (CT) of the head and electroencephalography (EEG) in patients having repeated convulsion and cerebral malaria (CM); ultrasonography of whole abdomen and specific test for hepatitis B and C in hepatic dysfunction and jaundice, and glucose-6-phosphate dehydrogenase (G6PD) enzyme level (kinetic method: G-SIX Kit; Crest Biosystems, Goa, India) for hemolysis. Blood culture was taken on brain–heart infusion broth in every patient who was having continuous high-grade fever >38.33°C for >24 h after admission. All the clinical symptoms were classified according to the WHO (Trampuz et al., 2003) criteria, and the involvement of two or more than two organs were considered as multiorgan dysfunction (MODS). Specific anti-malarial treatment was given in the hospital according to the WHO guidelines (Trampuz et al., 2003).

### Statistical Analysis

The data for all the assays are expressed as the mean ± standard deviation (SD) of three independent experiments done in triplicates. Student’s *t*-test was performed to calculate the *p-*values, where *p* < 0.05 was taken as significant. One-way ANOVA along with Tukey’s multiple comparisons test was performed to compare the three groups, with *p* < 0.05 was considered as significant.

## Results

### Infection of Erythrocytes With *P. falciparum* Reduces the Level of SphK-1

**FIGURE 1:**
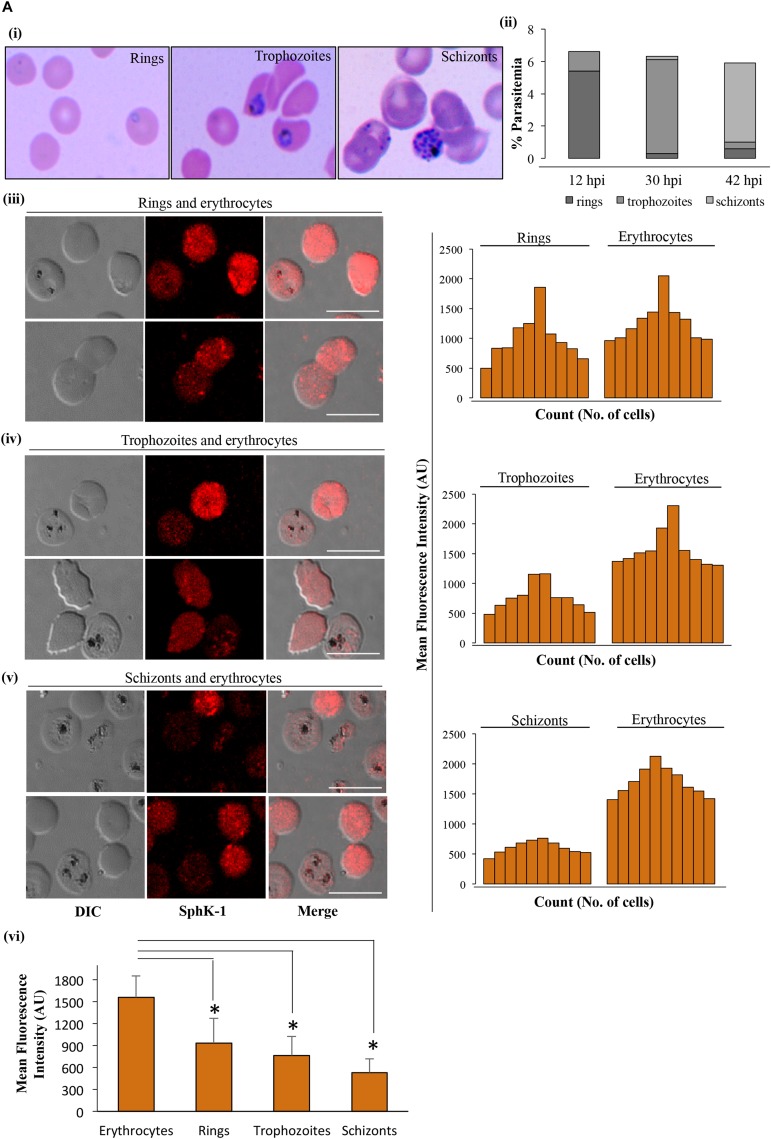
SphK-1 level decreases during infection with *Plasmodium falciparum* in erythrocytes. **(A) (i)** Giemsa-stained smears showing rings, trophozoites, and schizonts. Highly synchronized parasites at ring stage (10–12 hpi) were incubated in complete medium for the next 18 and 30 h, and stages were monitored by counting. **(ii)** Representative graph depicts the distribution of population with ring, trophozoite, and schizont stage-infected erythrocytes. **(iii–v)** Immunofluorescence assays (IFAs) were used to detect host SphK-1 level in uninfected and infected erythrocytes at different stages, namely, **(iii)** rings, **(iv)** trophozoites, and **(v)** schizonts using anti-SphK-1 rabbit antibody (1:1,000). A total of 10 cells were counted from each of the asexual cycle stages to plot the intensity graph. The level of host SphK-1 reduces as the parasite progresses from ring to schizont stage in the life cycle. **(vi)** Bar graphs represent the mean fluorescence intensity (MFI) and denote the SphK-1 levels in uninfected and infected erythrocytes, where 30 cells were used for calculation from uninfected erythrocytes and each of the asexual stages of infected erythrocytes for three biological replicates. Bar, 10 μm, (*n* = 3), ^∗^*p* ≤ 0.05.

Sphingosine kinase-1 catalyzes the phosphorylation of S1P from sphingosine in erythrocytes (Nakahara et al., 2012), and its level can be directly correlated with levels of IC or circulating S1P. IFA was performed to detect the level of SphK-1 in erythrocyte and parasite-infected erythrocyte. To elucidate the levels of SphK-1 in all intra-erythrocyte stages of *P. falciparum*, culture at 5–6% parasitemia with highly synchronized parasites at ring stage (10–12 hpi) was incubated in complete medium for a period of 18 and 30 h, and the stages were monitored by counting. For IFA, synchronous population of ring-, trophozoite-, and schizont-infected erythrocytes were used (Figures 1Ai,ii). The level of SphK-1 in uninfected and in parasite-infected erythrocytes at different asexual stages, namely, (iii) rings, (iv) trophozoites, and (v) schizonts was determined by measuring the intensity of SphK-1 in acquired images, and the representative MFI was calculated as described in the section “Materials and Methods.” Following comparative analysis, the SphK-1 level was found to be significantly lowered in parasite-infected erythrocytes than the respective uninfected controls. The level of host SphK-1 was reduced gradually as the asexual cycle progressed from ring to schizont stage (Figures 1Aiii–v). The average of MFI for the Sphk-1 level in rings, trophozoites, schizonts, and uninfected erythrocytes was plotted indicating significant difference in the levels of Sphk-1 (Figure 1Avi). This reduced level could be due to the degradation of host SphK-1 during parasite infection, as transcriptional regulation could not be accounted in erythrocytes due to lack of protein translation machinery.

### Infection of *P. falciparum* to Human Erythrocytes Decreases the Activity of SphK-1

**FIGURE 2:**
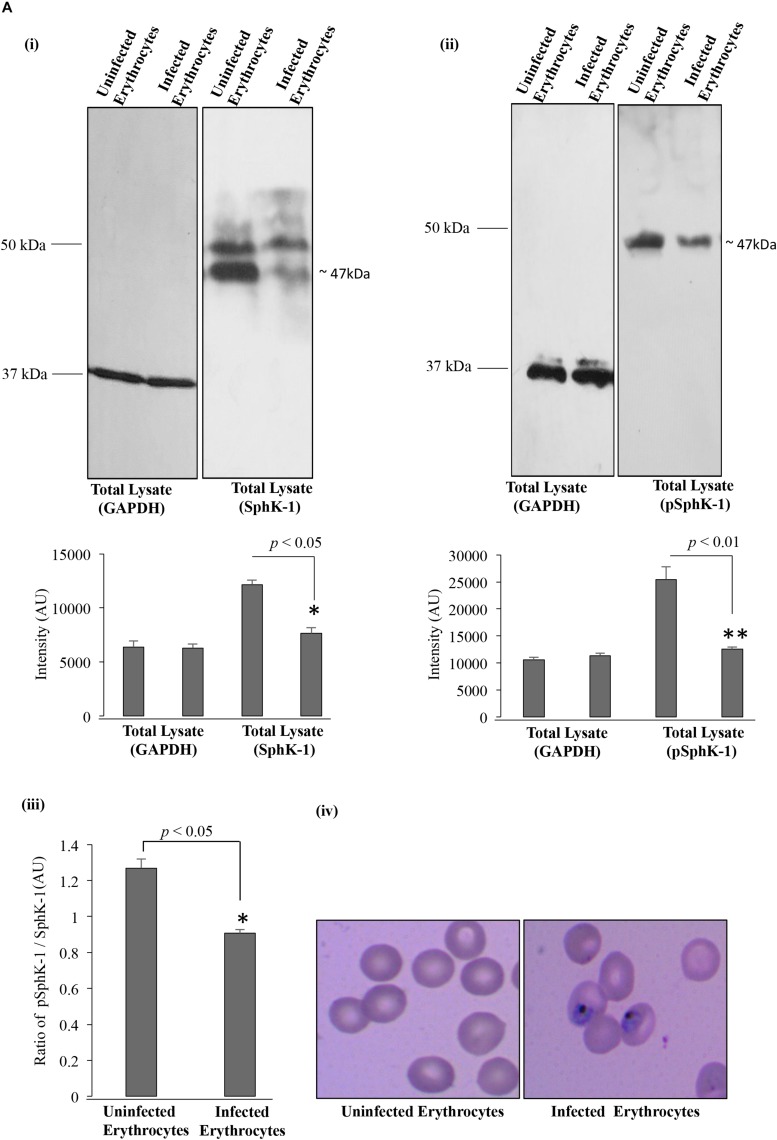
SphK-1 level and its phosphorylation decrease upon infection with *P. falciparum* in erythrocytes. **(A) (i)** Level of SphK-1 was detected by immunoblotting in uninfected and infected erythrocytes. Total cell lysates were separated on 10% SDS-PAGE and probed with anti-SphK-1 rabbit antibody (1:3,000). Total cell lysates probed with GAPDH was used as a loading control. In infected erythrocytes, SphK-1 level was lowered. The graph denotes intensity of bands in individual lanes. Three independent experiment have been performed, *n* = 3, ^∗^*p* ≤ 0.05. **(ii)** Phosphorylation status of SphK-1 during parasite infection was detected using immunoblotting. Total cell lysates of uninfected and infected erythrocytes were separated on 10% SDS-PAGE and probed with specific Phospho-SphK-1 (Ser225) rabbit antibody (1:1,000) against Phosphoserine 225. Total cell lysates probed with GAPDH was used as a loading control. In infected erythrocytes, SphK-1 phosphorylation was lowered. Graph denotes intensity of bands in individual lanes. Three independent experiments have been done, *n* = 3, ^∗∗^*p* ≤ 0.01. **(iii)** The graph represents the ratio of SphK-1 and pSphK-1 in parasite-infected and uninfected erythrocytes, ^∗^*p* ≤ 0.05. **(iv)** Giemsa-stained smears of uninfected and infected erythrocytes used for the immunoblotting are shown. p, phosphorylated.

In order to confirm the reduced level of host SphK-1 during parasite infection and its correlation to the decreased phosphorylation pattern, immunoblotting was performed for both SphK-1 and the phosphorylated form of SphK-1 (pSphK-1) in the total cell lysates of parasite-infected and uninfected erythrocytes. For this experiment, the parasitemia was maintained at 12–15% at the trophozoite stage. Respective lysates were probed with specific anti-SphK-1 and phospho-SphK-1 (Ser225) antibodies. Probing with anti-GAPDH antibody was done for use as the loading control. In line with the immunofluorescence detection, the host SphK-1 level was found to be reduced in parasite-infected erythrocytes compared to the uninfected erythrocytes. The band intensities in individual lanes of immunoblot were quantified by Image J to confirm the relative level of SphK-1 and pSphK-1. Interestingly, the phosphorylated form of host SphK-1 (pSphK-1) was also relatively decreased in parasite-infected erythrocytes (Figures 2Ai,ii). Band intensities for Sphk-1 and pSphk-1 in both uninfected and infected erythrocytes were normalized with values obtained from GAPDH. The relative ratio of normalized band intensity for SphK-1 and pSphK-1 was determined, and the ratio of pSphK-1 to SphK-1 intensity was represented as AU. pSphk-1/SPHK-1 ratio was found to be lowered in parasite-infected erythrocytes compared to the uninfected (Figure 2Aiii). Supportive Giemsa-stained images represented the uninfected and infected erythrocytes, which were used for the quantification of SphK-1 and pSphK-1 levels (Figure 2Aiv). These findings indicated a reduced level and phosphorylation-dependent activity of host SphK-1. Since S1P is synthesized from sphingosine by pSphK-1, reduced levels of host pSphK-1 during parasite infection might lead to deregulated S1P levels in plasma.

### *P. falciparum* Infection to Human Erythrocytes Led to Severe Reduction in S1P Levels

**FIGURE 3:**
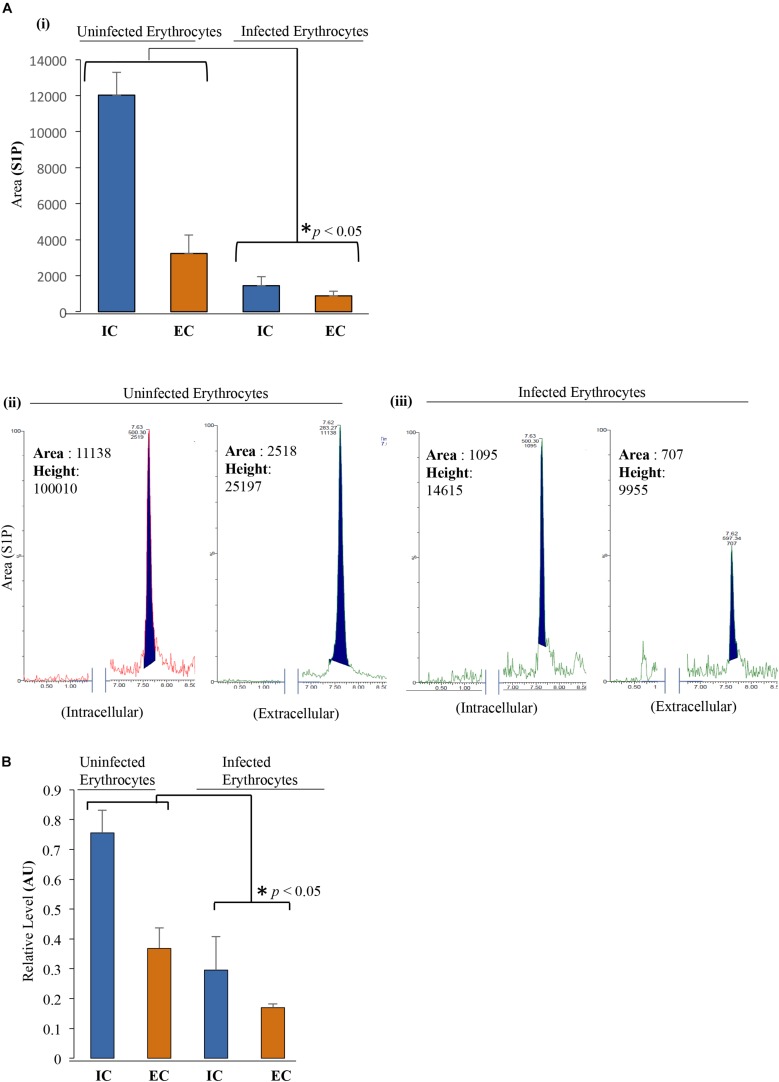
S1P levels decrease upon infection with *P. falciparum* in erythrocytes. **(A) (i)** LC/MS was used to quantify the total S1P levels, both in intracellular (IC) and extracellular (EC) uninfected erythrocytes and infected erythrocytes. Supernatant and cell pellet represent EC and IC S1P levels, respectively. Uninfected erythrocytes and infected erythrocytes were incubated in incomplete media followed by harvesting the supernatant and cell pellet by centrifugation at 1,500 × *g*. Lipid extraction from the samples was done according to the protocol mentioned under the section “Materials and Methods.” Bar graph represents the level of S1P in uninfected erythrocytes and infected erythrocyte IC as well as EC milieu. **(ii,iii)** Acquired S1P level represented by peak area and height highlighted in the MS spectra. Two biological replicates have been used for the experiment, *n* = 2, ^∗^*p* ≤ 0.05. The level of S1P is significantly low in infected erythrocytes compared to that in uninfected erythrocytes in both IC and EC environments of both the samples. **(B)** ELISA-based estimation was used to quantify the total S1P levels in both IC and EC of uninfected erythrocytes and/or infected erythrocytes as represented in the bar graphs. ELISA plate wells were pre-coated with S1P antibody followed by addition of supernatant and lysed pellet to measure the relative levels of S1P in EC and IC milieu. Three biological replicates have been used for the estimation of S1P; *n* = 3, ^∗^*p* ≤ 0.05.

In an effort to determine the role of SphK-1 level and its phosphorylation in regulating the S1P levels during malaria parasite progression, we determined the relative levels of S1P in IC and EC milieu of the erythrocytes through LC/MS and ELISA-based analyses. In this experiment, we have used 2.6 × 10^9^ cells/200 μl of packed volume of uninfected and infected erythrocytes. Trophozoite stage was used with 10–12% of parasitemia. Lipid extracts were prepared from *P. falciparum*-infected and/or uninfected erythrocytes and subjected to LC/MS analysis, as earlier described (Zhao and Xu, 2010). A characteristic peak at position 378.16 in the MS spectra was detected for S1P only as a control (Supplementary Figure 1). A similar peak of S1P was also detected in the IC as well as in the EC milieu of the erythrocytes (Supplementary Figures 2i,ii). Next, to compare the relative abundance of S1P in *P. falciparum*-infected and uninfected erythrocytes, we measured the S1P levels in the IC and EC milieu by LC/MS. The characteristic peak of S1P was also observed in the MS spectra at 378.16 for infected and uninfected erythrocytes (Supplementary Figures 2iii,iv). Further analysis revealed that S1P level was downregulated in the parasite-infected erythrocytes compared to the uninfected (Figure 3Ai). The peak areas and heights were obtained for S1P in the MS spectra for the uninfected erythrocytes [(IC) area: 11,138, height: 100,010; (EC) area: 2,518, height: 25,197] and for the infected erythrocytes [(IC) area: 1,095, height: 14,615; (EC) area: 707, height: 9,955] (Figure 3Aii,iii). Comparative analysis of S1P levels in the total IC + EC of both uninfected and infected erythrocytes was performed, and the difference in the S1P levels was found to be statistically significant (^∗^*p* < 0.05). To further validate the difference in S1P levels, ELISA-based S1P detection method was employed, and the comparison was done between infected and uninfected erythrocytes. Supernatant and lysed erythrocytes were added to micro ELISA plate pre-coated with S1P-specific antibody followed by probing with biotinylated antibody specific to human S1P. The results suggested that in parasite-infected erythrocytes S1P levels were significantly decreased compared to uninfected erythrocytes (Figure 3B). Further analysis of the relative S1P levels of total IC and EC, in the case of both uninfected and infected erythrocytes, demonstrated a significant reduction (^∗^*p* < 0.05), strongly supporting the observation from S1P levels detected in LC/MS (Figure 3Ai). These findings clearly indicated that decreased SphK-1 level and activity during the malaria infection significantly depletes the S1P levels that is in line with previous reports, wherein S1P has been shown to be downregulated during disease conditions (Punsawad and Viriyavejakul, 2017).

### Lower Levels of Circulating S1P Is Associated With Severity of *P. falciparum* and *P. vivax* Malaria Infection in Patients

**FIGURE 4:**
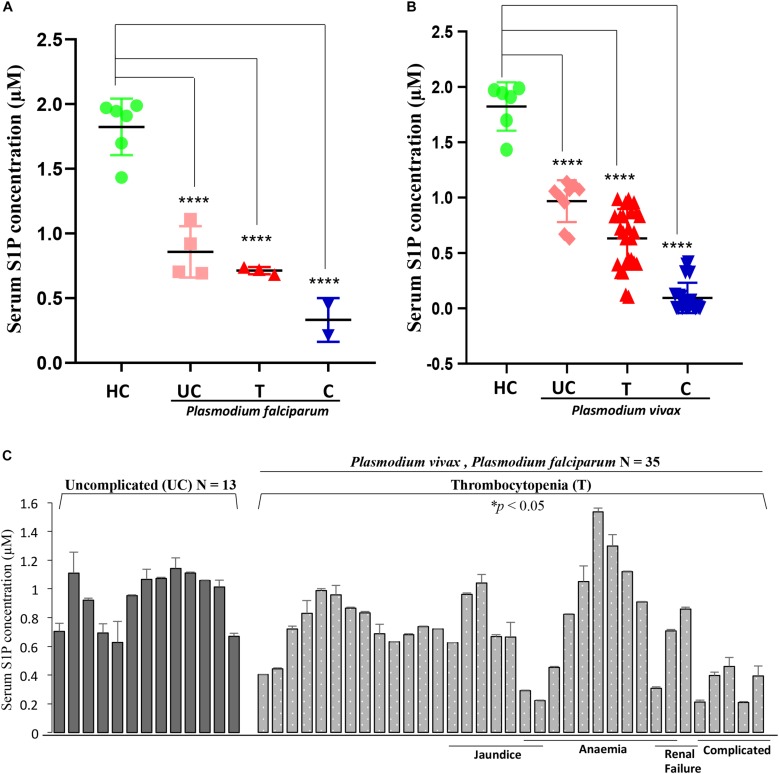
Serum S1P level in *P. falciparum*- and *P. vivax*-infected malaria patients decreases compared to the healthy individuals. **(A)** The representative scatter plot displays S1P levels (μM) in uncomplicated (UC), thrombocytopenia (T), and complicated cases (C) of *P. falciparum*-infected serum samples (*N* = 9) of malaria patients and healthy individuals (*N* = 6). One-way ANOVA followed by Tukey’s *post hoc* test revealed significant differences with *p*-values: ****p < 0.0001, ****p < 0.0001, ****p < 0.0001 (comparison with healthy individuals). **(B)** The scatter plot depicts serum S1P levels (μM) in uncomplicated (UC), thrombocytopenia (T), and complicated cases (C) of *P. vivax* malaria patients (*N* = 25) against the healthy individuals (*N* = 6). One-way ANOVA followed by Tukey’s *post hoc* test revealed significant differences with *p*-values: ****p < 0.0001, ****p < 0.0001, ****p < 0.0001 (comparison with healthy individuals). **(C)** The graph denotes serum S1P levels (in μM) in malaria patients with combined cases of thrombocytopenia (T) along with complicated (C), anemia (A), jaundice (J), and/or renal failure (RF) (*N* = 35) irrespective of *P. falciparum* and/or *P. vivax* infection versus uncomplicated patients (*N* = 13). The levels of S1P in all thrombocytopenia cases were found to be significantly lower than the uncomplicated malaria cases (*N* = 13). Student’s *t-*test was performed; * *p* < 0.05.

**FIGURE 5:**
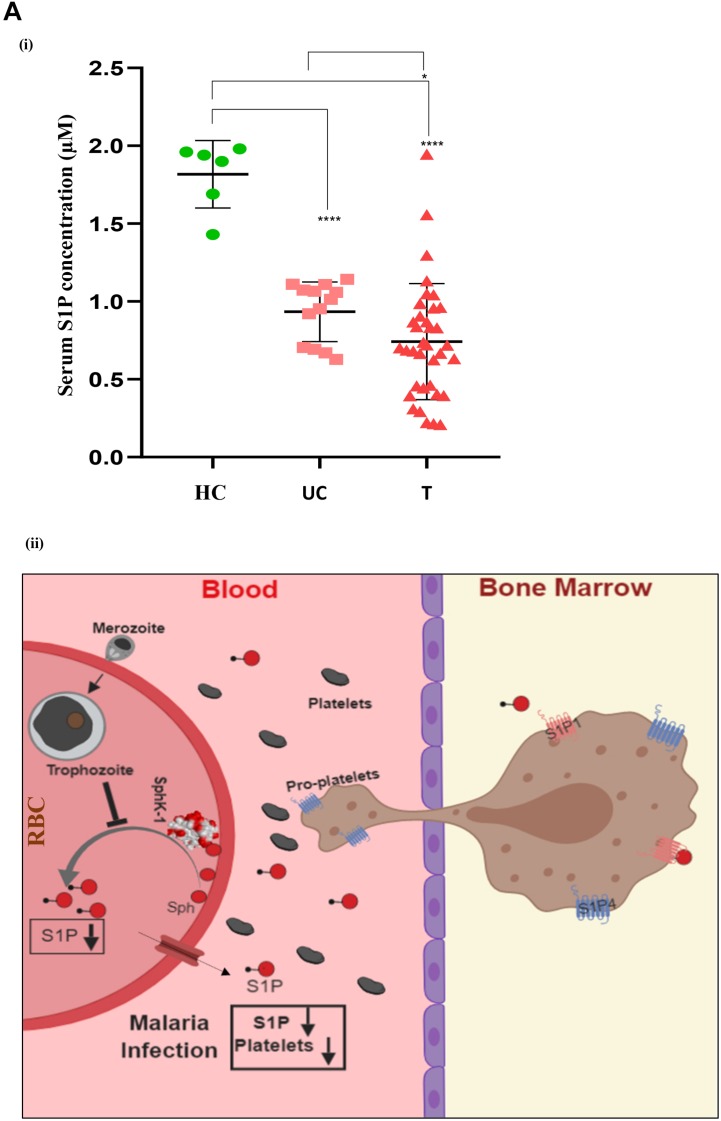
One-way ANOVA with Tukey’s multiple comparisons analysis among three groups, namely, healthy control (HC), uncomplicated malaria (UC), and thrombocytopenia patients (T). **(A) (i)** Comparative analysis among the groups, namely, HC (*N* = 6) and UC (*N* = 13), HC (*N* = 6) and T (*N* = 35), and UC (*N* = 13) and T (*N* = 35) was done with one-way ANOVA followed by Tukey’s *post hoc* test, which revealed significant differences with *p-*values: ****p < 0.0001, ****p < 0.0001, and *p < 0.05 respectively. **(ii)** Working model: role of erythrocytic SphK-1 in decreasing serum S1P and platelet levels. *P. falciparum* infection to human erythrocyte attenuates the erythrocytic SphK-1 by regulating its level and phosphorylation. In turn, SphK-1 modulates S1P production, hence, reducing its concentration in the blood. S1P gradient is altered during the malaria infection inhibiting the S1P/S1P receptor (S1P1 and S1P4) signaling pathway in the megakaryocytes. This results in decreased platelets shedding from the megakaryocytes and leading to clinical parameters like thrombocytopenia and anemia associated with disease severity.

In order to determine the serum concentrations of S1P and its association to disease severity in malaria, we have selected complicated and uncomplicated malaria patients, comprising both *P. falciparum* (*N* = 15)- as well as *P. vivax* (*N* = 36)-infected cases (Table 1). The control group was selected as healthy uninfected blood samples (*N* = 6), collected from the blood bank. Categorization of *P. vivax* and *P. falciparum* or mixed infection was done in all the patients as described in the section “Materials and Methods” and shown in Supplementary Figure 3. Circulating S1P levels in patient serum and healthy individuals were measured using ELISA-based analysis. Serum samples were added to a micro ELISA plate pre-coated with S1P-specific antibody, followed by probing with biotinylated antibody specific to human S1P. The S1P levels in patients suffering from *P. falciparum* (*N* = 9) and CM (*N* = 2/9) were found to be the lowest compared to the uncomplicated malaria (*N* = 4/9), thrombocytopenic cases (*N* = 3/9), and healthy controls (*N* = 6). Uncomplicated malaria and thrombocytopenic cases also showed lower levels of circulating S1P than the healthy controls. Student’s *t*-test was performed and the *p*-values detected as ^∗^*p* < 0.05, ^∗^*p* < 0.05, ^∗∗^*p* < 0.01 (comparison with healthy individuals) (Figure 4A). However, we also compared the S1P levels in *P. vivax* malaria patients (*N* = 25), wherein complicated malaria patients (*N* = 6/25) were found to have lowest circulating S1P concentration compared to uncomplicated malaria patients (*N* = 9/25), thrombocytopenia cases (*N* = 10/25), and healthy individuals (*N* = 6). Student’s *t*-test was performed and the *p*-values generated as ^∗^*p* < 0.05, ^∗^*p* < 0.05, ^∗∗^*p* < 0.01 (comparison with healthy individuals) (Figure 4B). Further, we detected the S1P levels in malaria patients with thrombocytopenia having clinical features of CM, anemia, jaundice, complicated and renal failure (*N* = 35), irrespective of *P. falciparum* and *P. vivax* infection. The levels of S1P in all thrombocytopenia cases were found to be significantly lower than those of the uncomplicated malaria cases (*N* = 13). Student’s *t*-test was performed; ^∗^*p* < 0.05 (Figure 4C). Further comparative analysis was performed using one-way ANOVA along with Tukey’s multiple comparisons test for three groups, namely, healthy control, uncomplicated malaria, and thrombocytopenia cases. The representative scatter plot showed the significant difference between *healthy control* and *uncomplicated malaria patients* (^****^p < 0.0001), *healthy control* and *malaria patients with* thrombocytopenia (^****^p < 0.0001), and between *uncomplicated malaria* and *malaria with thrombocytopenia* (^∗^*p* < 0.05) (Figure 5Ai). Altogether, these results indicated the relative decrease in serum levels of S1P in *P. falciparum* and *P. vivax* malaria patients compared to the healthy controls and found to be in line with the *in vivo* studies done earlier (Punsawad and Viriyavejakul, 2017). Here, based on our findings, we propose that a reduction in serum S1P is due to the corresponding decrease in host SphK-1 level and activity during malaria infection. Moreover, a significant S1P reduction was observed in infected samples diagnosed with clinical parameters like, thrombocytopenia along with other clinical manifestations such as anemia, jaundice, and/or renal failure, suggesting a strong correlation of S1P level with the disease severity (Table 1). This finding was in sync with previous reports, which showed a correlation of S1P levels with the disease severity in dengue, sepsis, etc. (Puneet et al., 2010; Gomes et al., 2014). These findings also suggested that parasite infection might affect the synthesis of S1P in serum leading to lower platelet production, which ultimately manifested as thrombocytopenia in malaria patients. This hypothesis has been described schematically in Figure 5Aii.

**TABLE 1:** Clinical data of the malaria patients and healthy controls showing the S1P level.

Clinical parameter	*P. falciparum*	*R. falciparum*	*P. vivax*	*P. vivax*
	monoinfection (*N* = 15)	SIP level (in μM) average ± SD	monoinfection (*N* = 36)	SIP level (in μM) average ± SD
Complicated malaria	2	0.33 ± 0.020	6	0.28 ± 0.027
Uncomplicated malaria	4	0.85 ± 0.069	9	0.96 ± 0.037
Thrombocytopenia	3	0.71 ± 0.003	10	0.736 ± 0.038
Jaundice + Thrombocytopenia	2	0.79 ± 0.048	3	0.79 ± 0.061
Jaundice + Anemia + Thrombocytopenia	2	0.22 ± 0.034	0	0
Anemia + Thrombocytopenia	1	0.45 ± 0.006	6	1.02 ± 0.037
Renal failure + Anemia + Thrombocytopenia	0	0	2	0.50 ± 0.042
Renal failure + Thrombocytopenia	1	0.78 ± 0.027	0	0
Renal failure + Jaundice + Thrombocytopenia	0	0	0	0

*Healthy control (*N* = 6) SIP level −1.82 μM. The data are presented as mean ± SD. SIP, sphingosine-1-phosphate.*

## Discussion

Infection of *P. falciparum* to human erythrocytes in blood stage progression involves multiple cycles of invasion and egress that modulates the host cell lipids (Gulati et al., 2015) that are involved in various lipid-dependent signaling pathways including IC signaling, protein trafficking, and hemoglobin degradation (Mamoun et al., 2010; Gulati et al., 2015). Synthesis of lipids and dependent pathways have been shown to be essential for the asexual parasite growth (Mamoun et al., 2010). Parasite-infected erythrocytes have at least twofold higher levels of lipids like phosphatidylglycerol (PG), acyl PG, lyso-phosphatidylinositol (LPI), bis (monoacylglycero) phosphate (BMP), monosialodihexosyl-ganglioside (GM3), diacylglycerol (DAG), and triacylglycerol (TAG) compared to the uninfected erythrocytes (Gulati et al., 2015). *P. falciparum* is capable enough to mediate the import of lipids like ceramide, sphingolipids, and LysoPC and utilize these lipid molecules to fulfill its own metabolic needs (Haldar et al., 1991; Gerold and Schwarz, 2001; Asahi et al., 2005). A recent report by Beri et al. (2019) highlighted that parasite-infected RBCs release sphingolipid metabolites, and alteration in the sphingolipid metabolism post infection might contribute toward change in RBC membrane dynamics. An additional study by Sana et al. (2013) also demonstrated a global metabolomic profiling of *P. falciparum*-infected erythrocytes that revealed modulation of various host lipids including S1P during parasite infection. Though these reports have suggested that the parasite invasion triggers alteration in sphingolipid metabolism in infected erythrocytes, there is still a lacuna that exists in the understanding of the possible molecular mechanism underlying the altered S1P metabolism during malaria pathogenesis.

To bridge the gap in understanding, we, for the first time, elucidated the regulation of S1P in malaria infection *in vitro.* SphK (isoforms SphK-1 and SphK-2) catalyzes the formation of the potent bioactive S1P that is produced and stored in various cell types including erythrocytes (Hänel et al., 2007), platelets (Schaphorst et al., 2015), endothelial cells (Pham et al., 2010), mast cells (Jolly et al., 2004), and macrophages (Xiong et al., 2013). However, erythrocytes have been considered as the main reservoir for S1P in the blood plasma (Hänel et al., 2007; Bode et al., 2010). Moreover, the liver is also engaged in modulating the plasma S1P content as it produces apolipoprotein M (ApoM), a chaperone for the S1P transport (Kleuser, 2018). S1P acts as a signaling molecule mediating regulation of various cellular processes by activating a family of G protein-coupled receptors [GPCRs, also termed as sphingosine-1-phosphate receptor 1–5 (S1P_1__–__5_)] (Pyne et al., 2016). It is noteworthy that SphK-1 resides in the cytosol of the erythrocytes (Pitson et al., 2003) and its phosphorylation are essential for its activity. Notably, SphK-1 selectively binds to phosphatidylserine in the membrane and mediates the synthesis of S1P at the cell membrane in close proximity to its substrate sphingosine (Delon et al., 2004; Pitson, 2011). Additionally, phosphatidylserine has shown to increase the SphK-1 activity by a dose-dependent manner (Olivera et al., 1996). Indeed, the exposure of PS to the outer leaflet of the plasma membrane (and therefore its disappearance from the inner leaflet) may well be a mechanism for the reduced activity of SphK1. In macrophages, a decrease in SphK-1 phosphorylation was reported during leishmaniasis (Arish et al., 2018), and SphK-1 inhibition was also reported in dengue virus infection in HEK-293 cells (Gomes et al., 2014). Plausibly, SphK activity and S1P signaling have been associated with several infections, including acute dengue infection (Gomes et al., 2014), sepsis (Puneet et al., 2010), chronic hepatitis C infection (Ikeda et al., 2010), obesity (Kowalski et al., 2013), etc. In case of viral infection, it has been reported that a non-structural protein NS3 from the bovine viral diarrhea virus inhibits the catalytic activity of SphK-1 (Yamane et al., 2009). However, it is yet not established what happens to the role of SphK-1 during *P. falciparum* infection. In other systems, Sphk1 has been shown to be phosphorylated by ERK1/2 (Pitson et al., 2003) and PKC (Johnson et al., 2002); interestingly, both host erythrocyte MEK (a component of the ERK pathways) (Sicard et al., 2011) and PKC (Hall et al., 1997) have been implicated in *Plasmodium* infection. It would be of interest to determine whether Sphk1 is a mediator of these pathways in infected erythrocytes.

Toward this, we report for the first time that host-mediated SphK-1 level and its phosphorylation status decrease during malaria infection (Figures 1A, 2A) that could be the possible reasons underlying the reduced levels of S1P in the sera of malaria-infected patients, in line with the previous studies (Pitson, 2011; Punsawad and Viriyavejakul, 2017). LC–MS/MS and ELISA-based determination of S1P levels *in vitro*, from the IC and EC milieu of both infected and uninfected erythrocytes, authenticated that *P. falciparum* infection drastically reduced the S1P pool (Figure 3), which strongly corroborated our laboratory findings from clinical studies, wherein the S1P level was drastically decreased in uncomplicated and complicated malaria along with thrombocytopenia in *P. falciparum*- and *P. vivax-*infected cases (Figure 4A). Earlier, reduced S1P level in malaria has been associated with clinical parameters like parasite count, platelet count, hemoglobin, and hematocrit levels, in relation with the severity of the disease (Punsawad and Viriyavejakul, 2017). Moreover, in megakaryocytes, S1P interacts with its receptors, S1P_1_ and S1P_4_, to trigger thrombopoiesis (Golfier et al., 2010; Zhang et al., 2012). Circulating S1P has also been known to maintain its gradient between vascular and non-vascular compartments, and modulation of this could result in attenuation of platelet production and their release into the circulation (Schwab et al., 2005; Hla et al., 2008). In our study, we have established a correlation of S1P regulation between clinical and laboratory findings of the malaria patients with clinical parameters like CM, uncomplicated malaria, thrombocytopenia, renal failure, jaundice, and/or anemia (Figure 4 and Table 1). Our study was strongly supported by a previous report that showed loss of S1P gradient in the circulation can result in a profound thrombocytopenia due to detrimental effects of attenuated proplatelet formation and fragmentation (Zhang et al., 2013). Since the circulating S1Ps are prerequisite for the growth of proplatelet strings in the blood stream and the shedding of platelets into the circulation, we assumed that loss of S1P levels can be linked to thrombocytopenia in malaria cases, which might be due to the defective proplatelet formation, a molecular consequence of altered S1P levels. Further, our study demonstrated low levels of SphK-1 and its reduced activity in infected erythrocytes that could be strongly correlated with the reduced S1P production *in vitro*. These results are in sync with our clinical data, which showed that patients suffering from severe forms of malaria represent diminished S1P levels. Based on our findings, we propose a regulation of host SphK-1 as a factor for reduced S1P levels during parasite infection, which might lead to attenuated thrombopoiesis via S1P_1_ and/or S1P_4_ signaling pathways, thereby leading to thrombocytopenia (Figure 5).

In this study, CM patients additionally diagnosed with thrombocytopenia, jaundice, anemia, and/or renal failure showed significantly decreased levels of circulating S1P (^∗^*p* < 0.01) proposing the severity of infection in correlation to altered S1P levels. Thus, S1P signaling has emerged as a novel therapeutic target, as many studies have acknowledged the fact that S1P-mediated signaling pathways play a critical role in numerous infectious disease manifestations (Maceyka et al., 2012). Hence, the modulation of S1P signaling pathways might provide a breakthrough to present alternative targets for drug development. For example, chemically synthesized sphingolipid derivatives, which mimic the biological properties of natural lipids can be used for investigating highly complex sphingolipid metabolism by rapid and selective “click chemistry” using sensitive tags like fluorophores (Fink and Seibel, 2018). To summarize, host SphK-1 level is reduced as the parasite asexual stage progresses with substantial decrease in its phosphorylation. Thus, serum S1P levels in *P. falciparum* and *P. vivax* malaria patients are also decreased, in line with the previous reports suggesting an altered S1P signaling during malaria infection implicating its severity (Punsawad and Viriyavejakul, 2017). Thrombocytopenia and anemia are associated with decreased S1P level during malaria infection. Hence, these findings can prove to be beneficial to open up new ventures of therapeutic possibilities to counteract the global problem of malaria infection.

## Data Availability Statement

The datasets generated for this study are available on request to the corresponding author.

## Ethics Statement

The studies involving human participants were reviewed and approved by the Hospital Ethical Committee (Department of Medicine, Sardar Patel Medical College and associated group of Hospitals, Bikaner, Rajasthan, India No. F. (Acad) SPMC/2003/2395). The patients/participants provided their written informed consent to participate in this study.

## Author Contributions

SS conceived and designed the research. RS performed the research. SS and SP analyzed the data. RS, SP, and SS conducted the lipid extraction and estimation experiments. MS and RS performed the microscopy experiments. SS, RS, and SP wrote the manuscript. AD was involved in the clinical study design. PB was involved in the sample processing. DK and SK were involved in classifying the clinical symptoms and coordinating sample collection.

## Conflict of Interest

The authors declare that the research was conducted in the absence of any commercial or financial relationships that could be construed as a potential conflict of interest.
