# Supplementary material for: Reduction of Sphingosine Kinase 1 Phosphorylation and Activity in Plasmodium-Infected Erythrocytes
Source: Front Cell Dev Biol. 2020 Mar 3;8:80. doi: 10.3389/fcell.2020.00080 (PMC7062701; doi:10.3389/fcell.2020.00080)
Supplement: Supplementary file 1 [file Data_Sheet_1.pdf]

Supplementary file

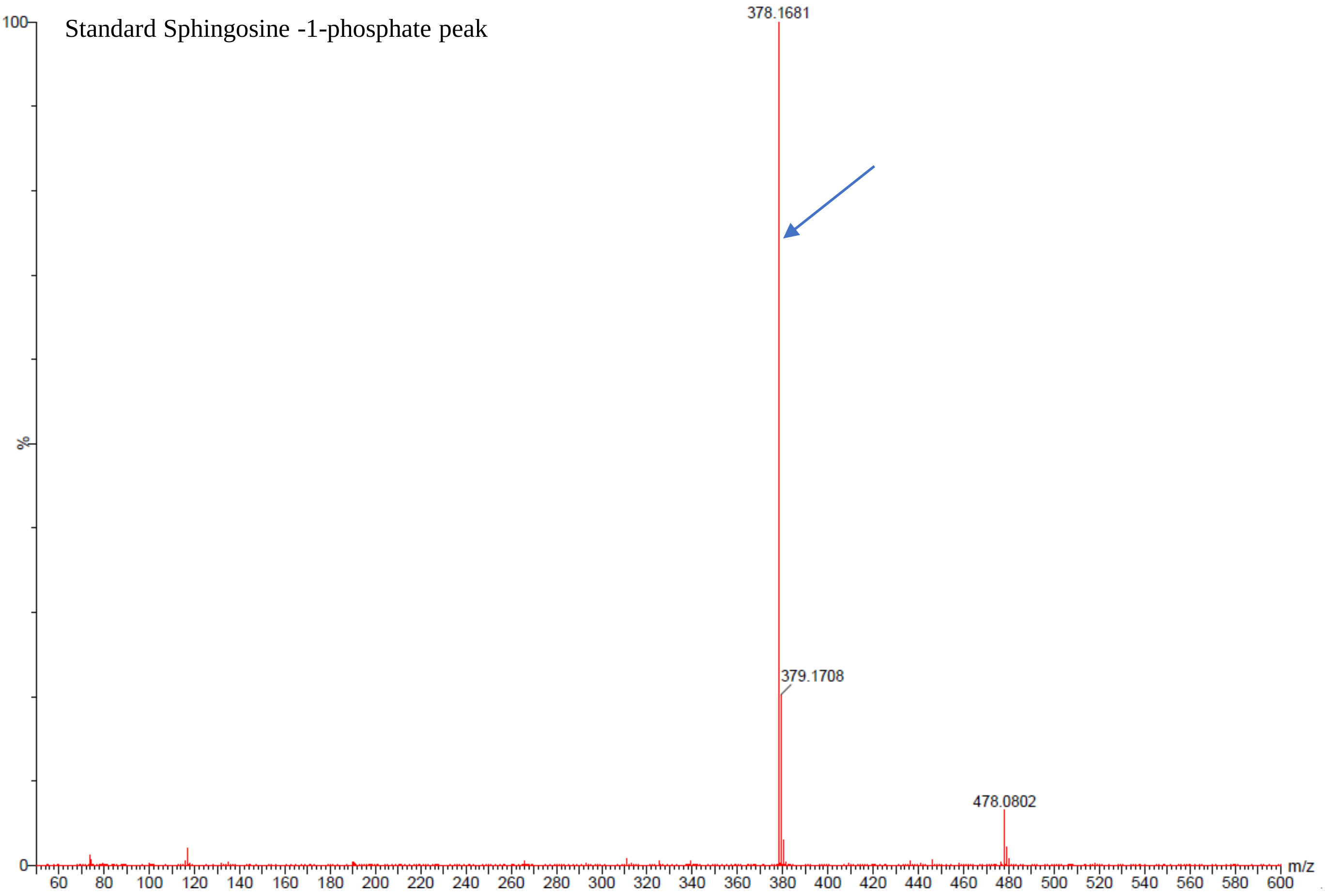

Supplementary Figure 1. Characteristic peak of S1P in MS spectra. S1P in methanol was subjected to LC/MS analysis and generated a characteristic peak at position 378.16 acquired in MS spectra.

Figure 2 (i)

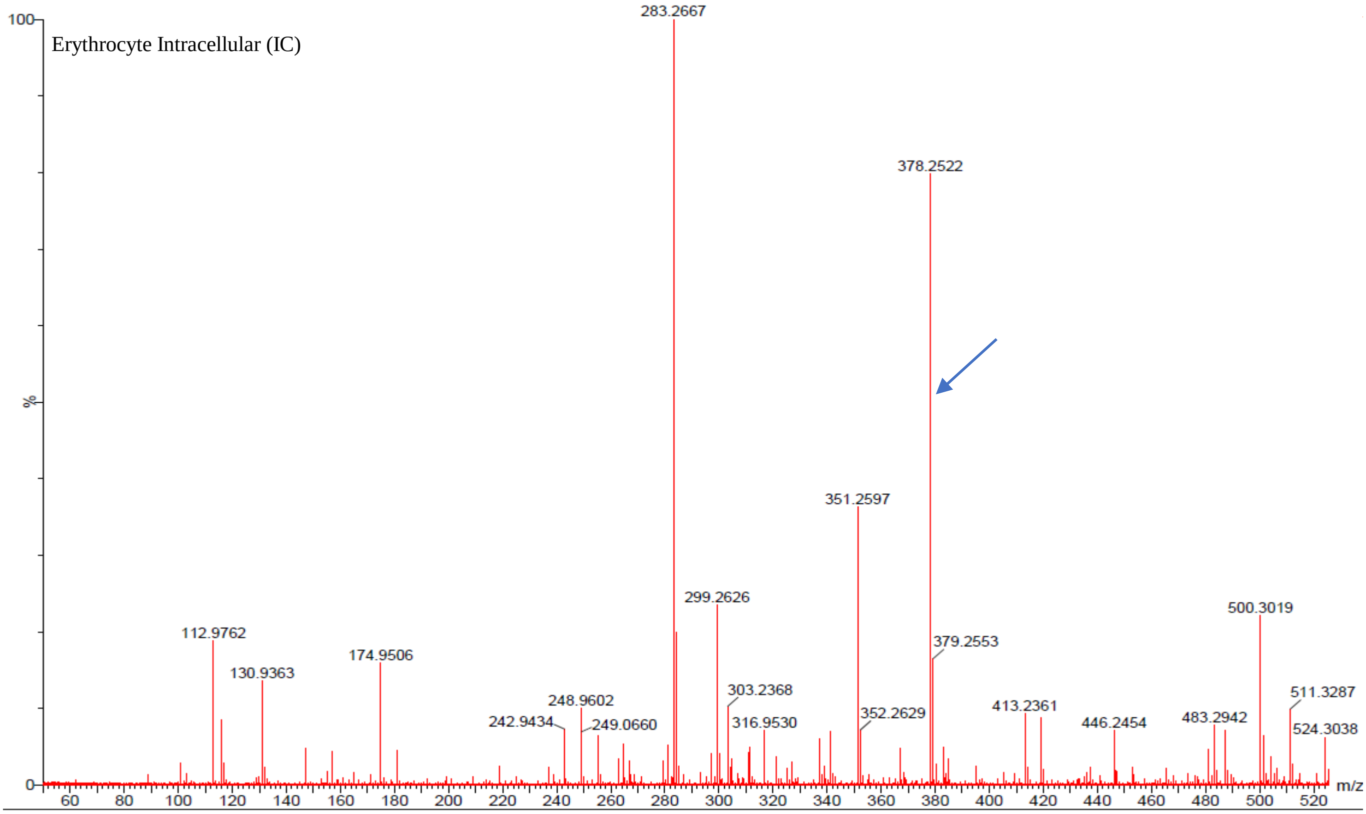

Erythrocyte Intracellular (IC)
100
%
0
283.2667
378.2522
351.2597
299.2626
500.3019
379.2553
112.9762
174.9506
130.9363
303.2368
511.3287
248.9602
413.2361
352.2629
242.9434
249.0660
316.9530
446.2454
483.2942
524.3038
60
80
100
120
140
160
180
200
220
240
260
280
300
320
340
360
380
400
420
440
460
480
500
520
m/z

(ii)

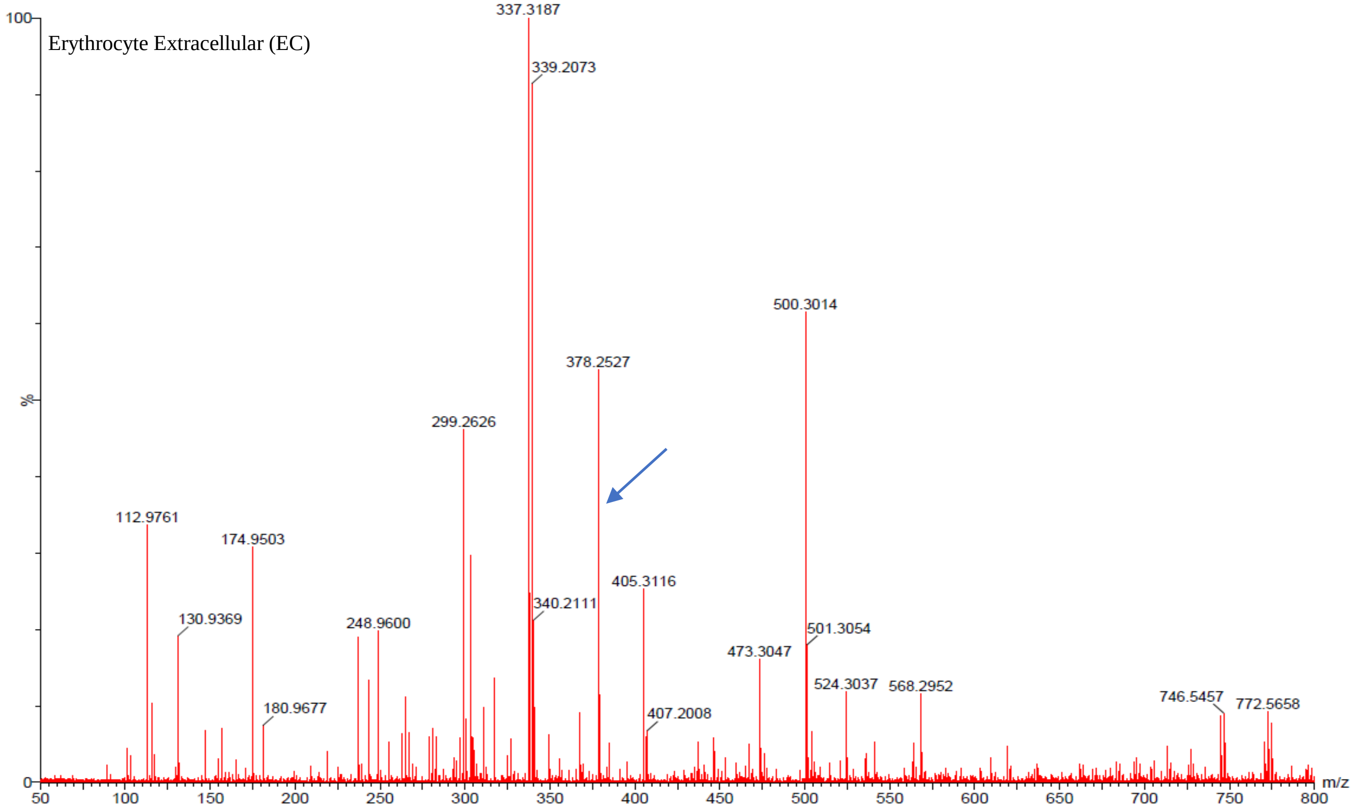

Erythrocyte Extracellular (EC)
100
%
0
337.3187
339.2073
500.3014
378.2527
299.2626
112.9761
174.9503
405.3116
340.2111
130.9369
248.9600
501.3054
473.3047
524.3037
568.2952
180.9677
746.5457
772.5658
407.2008
50
100
150
200
250
300
350
400
450
500
550
600
650
700
750
800
m/z

(iii)

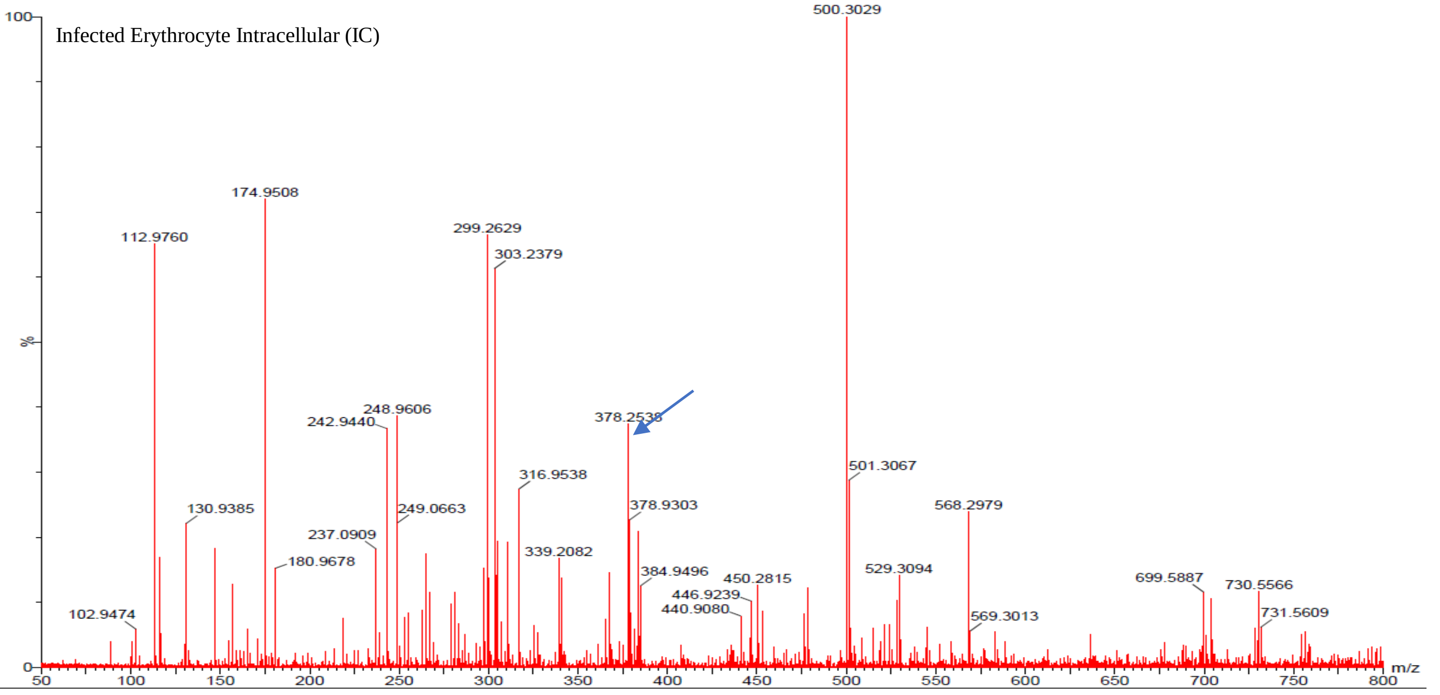

(iv)

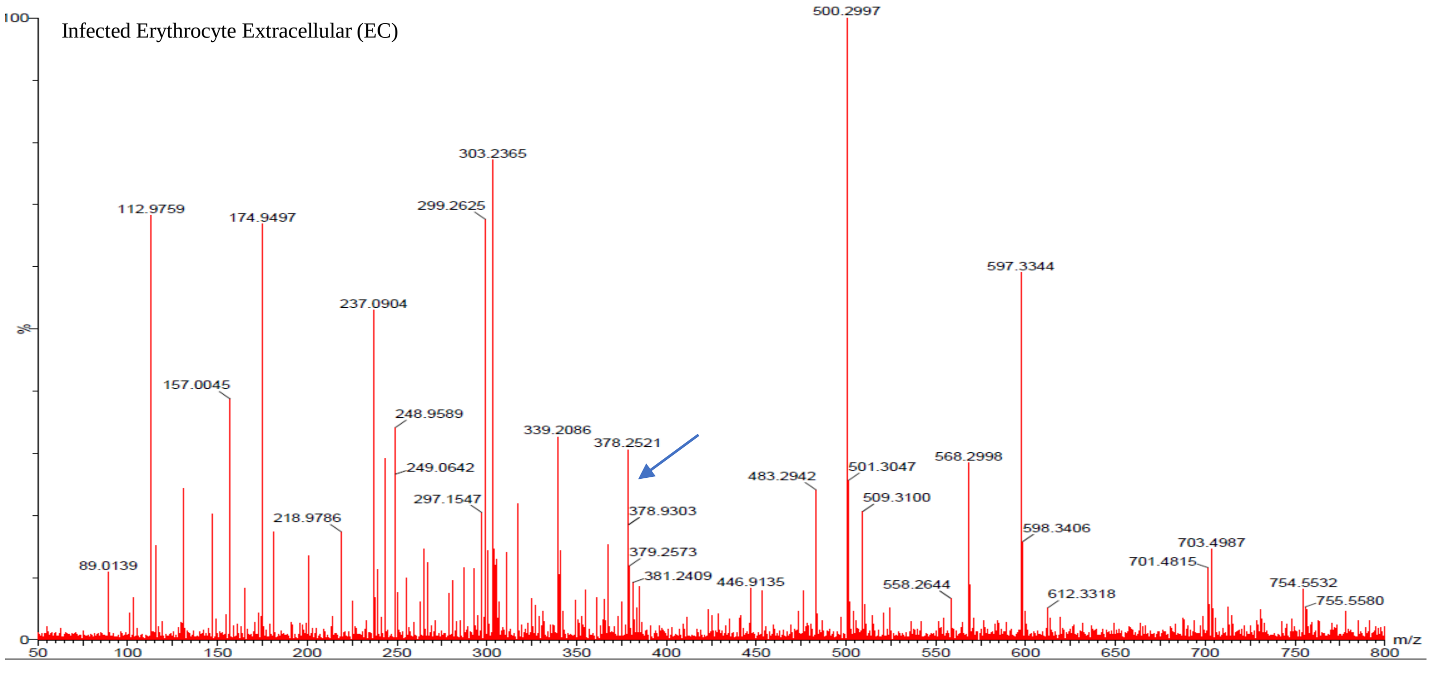

Supplementary Figure 2 (i)-(iv). S1P detection in IC and EC milieu of parasite infected and uninfected erythrocytes. Lipids were extracted from supernatant and lysed cells from parasite infected and uninfected erythrocytes. The extracted lipids were subjected to LC/MS analysis for S1P detection. S1P characteristic peaks were detected at position 378.25 in the MS spectra for IC as well as EC milieu of parasite infected and uninfected erythrocytes.

Figure 3

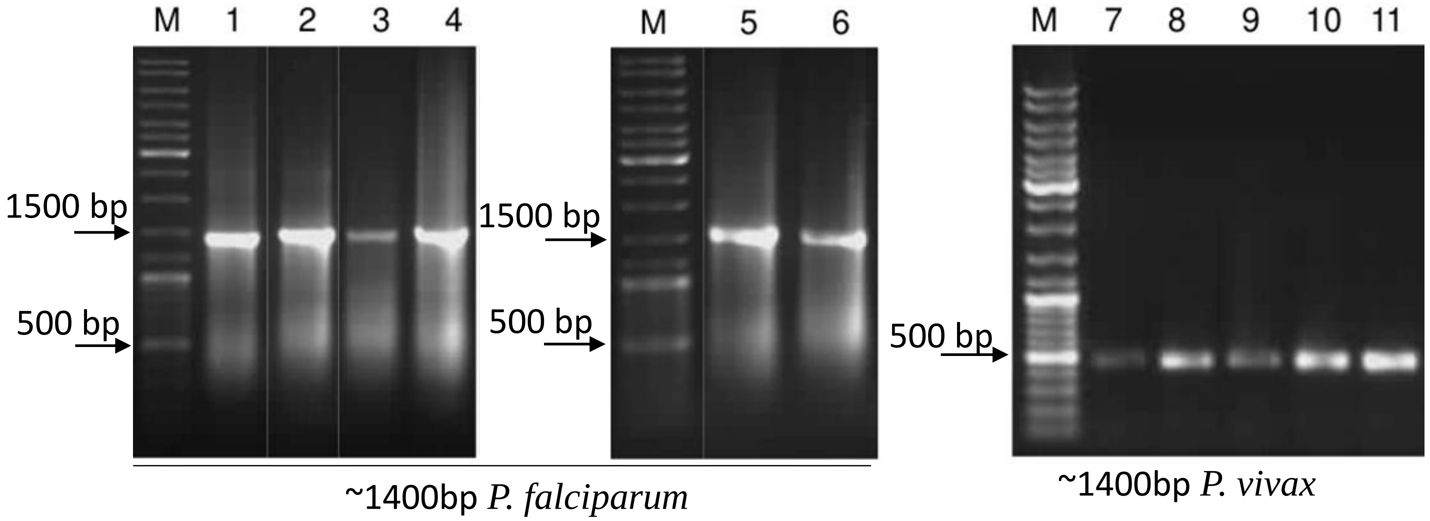

Supplementary Figure 3. Representative gel pictures of 18S Multiplex PCR performed on patient samples. Lane 1-6 correspond to *P. falciparum* positive samples showing band at ~1400bp; Lane 7-11 correspond to *P. vivax* positive samples showing band at ~500bp; M, 1kb DNA ladder (SM#0331; Thermo Scientific GeneRuler DNA Ladder Mix)
